# Natural History of *Alternaria* Sensitisation and Association With Asthma and Rhinitis From Childhood to Adulthood

**DOI:** 10.1111/all.70394

**Published:** 2026-05-18

**Authors:** Mohammad Leily, Hongmei Zhang, Graham Roberts, Pandurangan Vijayanand, Magnus Borres, Eva Södergren, Ramesh J. Kurukulaaratchy, S. Hasan Arshad

**Affiliations:** ^1^ The David Hide Asthma and Allergy Research Centre Newport UK; ^2^ Clinical and Experimental Sciences, Faculty of Medicine University of Southampton Southampton UK; ^3^ Respiratory Biomedical Research Centre University Hospital Southampton Southampton UK; ^4^ Division of Epidemiology, Biostatistics, and Environmental Health Sciences, School of Public Health University of Memphis Memphis Tennessee USA; ^5^ La Jolla Institute for Immunology La Jolla California USA; ^6^ Department of Women's and Children's Health Uppsala University Uppsala Sweden; ^7^ Thermo Fisher Scientific Phadia AB Uppsala Sweden; ^8^ Thermo Fisher Scientific Global Medical and Scientific Affairs Uppsala Sweden

**Keywords:** allergic sensitisation, *Alternaria*, asthma, IgE, rhinitis

## Abstract

**Background:**

Sensitisation to *Alternaria* is clinically important in asthma and rhinitis, but its life‐course patterns and optimal diagnostic markers are unclear. We aimed to characterise *Alternaria* sensitisation trajectories from childhood to adulthood; we compared skin prick testing (SPT), whole‐extract specific IgE and rAlt a 1, and assessed their longitudinal association with asthma and rhinitis in the Isle of Wight birth cohort.

**Methods:**

Participants were assessed at 4, 10, 18 and 26 years. *Alternaria* sensitisation was measured by SPT (all ages) and serum IgE to whole *Alternaria* extract and rAlt a 1 (10, 18, 26 years). “Any Alternaria” denoted positivity to any assay. Alternaria, asthma and rhinitis trajectories (10–18–26 years) were classified as Never, Any positive or Persistent. Associations used χ^2^ trend tests and Poisson regression with robust errors.

**Results:**

Among 434 participants with complete *Alternaria* data, trajectories were Never 84.1%, Any 11.1% and Persistent 4.8%. *Alternaria* trajectories showed graded associations with asthma, and more strongly with rhinitis (trend *p* < 0.001 for both), with highest risks for persistent asthma and rhinitis. Across ages, rAlt a 1 discriminated asthma risk better than whole‐extract in youth, whereas effects converged by 26 years. For rhinitis, effect sizes were similar across assays and ages. Assay agreement strengthened with age, becoming substantial by 18 years and near‐perfect by 26 years.

**Conclusions:**

*Alternaria* sensitisation consolidates from late adolescence and, when persistent, tracks with persistent asthma and rhinitis. Longitudinal analysis shows rAlt a 1 discriminates asthma risk better than whole‐extract in youth, while assays are largely interchangeable in adulthood.

## Graphical abstract

We aimed to characterise *Alternaria* sensitisation trajectories from childhood to adulthood; we compared SPT, whole‐extract sIgE and rAlt a 1, and assessed their longitudinal association with asthma and rhinitis in the Isle of Wight birth cohort. Alternaria sensitisation in the Isle of Wight Birth Cohort emerges mainly in late adolescence and, when persistent, tracks with persistent asthma and rhinitis from ages 10–26. Component‐resolved rAlt a 1 better discriminates asthma risk than whole‐extract sIgE in youth, while SPT and IgE‐based assays converge and are largely interchangeable by adulthood. AIC, Akaike Information Criterion; CI, confidence interval; *N*, number; *p*, *p*‐value; rAlt a 1, recombinant Alt a 1; RR, risk ratio; sIgE, specific immunoglobulin E; SPT, skin prick test.

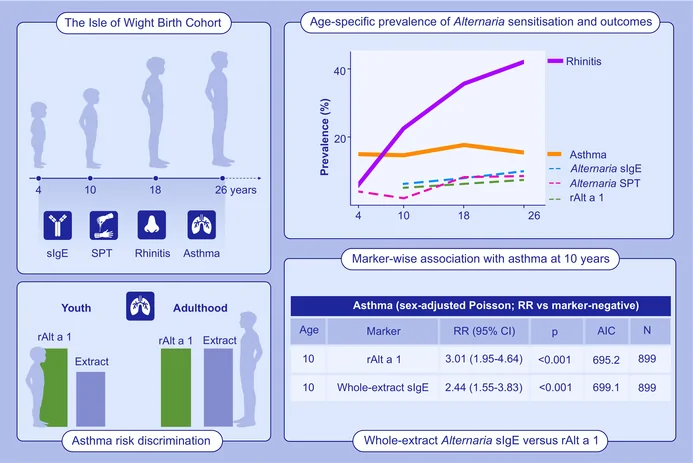

**Abbreviations**
BHR: bronchial hyper‐responsiveness
CI: confidence interval
CRD: component‐resolved diagnostics
DRS: dose–response slope
FEF25‐75: forced mid‐expiratory flow
FeNO: Fractional exhaled nitric oxide
FEV_1_
: forced expiratory volume in 1 s
FVC: forced vital capacity
IgE: immunoglobulin E
IOWBC: Isle of Wight Birth Cohort
IOWBC: rAlt a 1, recombinant Alt a 1
ISAAC: International Study of Asthma and Allergies in Childhood
RR: risk ratio
sIgE: serum specific IgE
SPT: skin prick test

## 1 Introduction

Sensitisation to fungal aeroallergens is common and clinically important in respiratory allergy [1, 2]. Among these, *Alternaria* spp. are ubiquitous outdoors and peak in late summer/early autumn; their spores are small, numerous, and capable of reaching the lower airways [1, 3, 4]. Cross‐sectional and time‐series studies link ambient *Alternaria* counts with emergency visits for asthma, epidemic “thunderstorm” asthma, and severe or near‐fatal exacerbations [1, 5, 6, 7]. At the individual level, *Alternaria* sensitisation associates with asthma and allergic rhinitis in population surveys and birth cohorts, and with airway hyper‐responsiveness even among non‐asthmatics [8, 9, 10, 11, 12]. *Alternaria* sensitisation has also been linked to more severe rhinitis and asthma phenotypes, including greater symptom burden, increased airway hyper‐responsiveness and higher exacerbation risk [3, 6, 7]. Skin‐prick testing (SPT) and serum specific IgE (sIgE) to Alternaria are established measures of allergic sensitisation that are widely used in clinical practice and epidemiological studies; when interpreted alongside symptoms and exposure, they provide clinically useful evidence supporting IgE‐mediated allergy [2].

Recombinant, component‐resolved diagnostics (CRD) quantify IgE to individual allergens and their component proteins and can help interpretation of a positive test using SPT and whole‐extract sIgE due to cross‐reactive proteins. They also improve standardisation and minimise variability related to allergen extract composition [13]. rAlt a 1, the major *Alternaria alternata* allergen, is a specific marker of primary *Alternaria* sensitisation and may improve diagnostic precision compared with whole‐extract testing [14, 15]. CRD may therefore enhance risk stratification compared with whole‐extract assays. Accordingly, we evaluate rAlt a 1 alongside whole‐extract sIgE and SPT at 10, 18 and 26 years.

Despite this body of evidence, two gaps remain. First, most reports are cross‐sectional [1, 2, 8, 9, 10, 11, 12], far fewer examine how *Alternaria* sensitisation evolves from late childhood through adolescence into early adulthood, a period when atopic profiles and respiratory phenotypes may emerge, remit, or persist [16, 17, 18]. Secondly, the clinical significance of *Alternaria* trajectories such as transient versus persistent sensitisation has not been evaluated alongside equally longitudinal disease trajectories for asthma and rhinitis across the same ages. Clarifying these life‐course patterns matters for risk stratification, counselling, and the timing of preventive strategies, including allergen avoidance and immunotherapy [19, 20].

The Isle of Wight Whole‐Population Birth Cohort (IOWBC) offers repeated SPT/sIgE assessments and harmonised respiratory phenotyping at ages 4, 10, 18 and 26 years, with consistent protocols and high follow‐up [21]. Leveraging these data allows a rigorous comparison of sensitisation trajectories with clinically relevant asthma and rhinitis courses across the transition from adolescence to adulthood.

### 1.1 Objectives

1. Describe the prevalence and natural history of *Alternaria* sensitisation trajectories from ages 4, 10, 18 and 26.
2. *Compare diagnostic modalities by evaluating the relative and joint predictive value of whole‐extract sIgE and rAlt a 1 (*vs *SPT) for asthma and rhinitis at each age, including model‐fit indices, to inform pragmatic testing strategies*.
3. Examine associations between *Alternaria* trajectories and asthma/rhinitis trajectories, including overall association and trend.
4. Estimate sex‐adjusted relative risks for asthma and rhinitis at age 26 comparing *Alternaria* trajectory groups with the never‐sensitised group.

Together, these analyses test whether the timing and persistence of *Alternaria* sensitisation meaningfully track with – and may help to inform – the life‐course expression of asthma and rhinitis in the general population.

## 2 Methods

### 2.1 Study Design and Population

The IOWBC was established in 1989 to investigate the natural history of asthma and allergic disease in an unselected community sample [21]. Of 1536 births (January 1989–February 1990), 1456 infants were enrolled with parental consent for longitudinal follow‐up. Cohort assessments were undertaken at ages 1, 2, 4, 10, 18 and 26 years. Parents completed standardised questionnaires up to age 10; participants self‐completed at ages 18 and 26. Ethical approval was granted at inception (Approval No. 05/89; 22 August 1988) with renewals for each follow‐up; written informed consent/assent was obtained at every stage.

### 2.2 Sensitisation Assessment

At ages 4, 10, 18 and 26 years, skin‐prick testing (SPT) was performed to a standard inhalant panel including grass and tree pollens, house dust mite, cat and dog dander, and common moulds (including *Alternaria*), using positive (histamine) and negative (saline) controls; a wheal ≥ 3 mm greater than the negative control was considered positive [22]. At ages 10, 18 and 26, serum specific IgE (sIgE) to whole Alternaria extract and to recombinant Alt a 1 were quantified using ImmunCAP (Thermo Fisher Scientific, Uppsala, Sweden); positivity was defined as ≥ 0.35 kUA/L (conventional clinical threshold) [23, 24, 25] For each age, a participant was classified as *Alternaria*‐sensitised if any of SPT, whole‐extract sIgE or Alt a 1 was positive. Detailed sIgE laboratory methods are provided in the Methods S1.

### 2.3 Respiratory Outcomes

Asthma and rhinitis were ascertained at each age using instruments based on the International Study of Asthma and Allergies in Childhood (ISAAC) methodology [26]:

Current asthma: physician diagnosis of asthma with current wheeze and/or current treatment for wheeze/asthma.

Rhinitis: typical nasal and/or ocular symptoms when not having a cold.

Primary endpoints were: (i) asthma at age 26; (ii) rhinitis at age 26; (iii) persistent asthma across ages 10, 18 and 26 (positive at all three ages); and (iv) persistent rhinitis across ages 10, 18 and 26 (positive at all three ages).

### 2.4 Lung Function, FeNO and Bronchial Hyper‐Responsiveness

At ages 10, 18 and 26 years, spirometry was performed according to contemporaneous guidelines [27], with the best of reproducible manoeuvres retained. Forced expiratory volume in one second (FEV_1_) and forced vital capacity (FVC) were expressed as percentages of predicted values based on age‐, sex‐ and height‐specific reference equations, and the FEV_1_/FVC ratio was calculated at each age.

Fractional exhaled nitric oxide (FeNO) was measured at 18 and 26 years using standardised online techniques [28]; values were approximately log‐normally distributed and were therefore analysed on the natural‐log scale. For presentation, sex‐adjusted geometric mean FeNO and 95% confidence intervals (CIs) were obtained by exponentiating model‐based estimates and their CIs.

At ages 10 and 18 years, bronchial hyper‐responsiveness (BHR) was assessed by standardised bronchial provocation testing following American Thoracic Society guidelines [29]. Testing used an inhaled methacholine challenge (after saline) with incremental concentrations from 0.0625 to 16 mg/mL, with spirometry measured 1 min after each dose. BHR was summarised using a dose–response slope (DRS) derived from the change in FEV_1_ across incremental doses of bronchoconstrictor [30], with larger values indicating greater hyper‐responsiveness. Because DRS was markedly right‐skewed, we analysed the log‐transformed squared DRS (log[DRS^2^]) at each age; higher log[DRS^2^] reflects greater BHR.

### 2.5 Trajectory Definitions

With three time points (10, 18 and 26 years), there are eight possible *Alternaria* sensitisation patterns. To reduce sparse cells and reflect clinically meaningful patterns, *Alternaria* sensitisation and each disease outcome (asthma, rhinitis) were summarised over ages 10–18–26 in a prespecified three‐group schema:

1. Never—negative at all three ages;
2. Any positive (non‐persistent)—positive at one or two ages;
3. Persistent—positive at all three ages.

Trajectory distributions for *Alternaria* were described among participants with complete *Alternaria* data at all three ages; trajectory distributions for asthma and rhinitis were described among participants with complete outcome data at all three ages. All analyses used complete‐case denominators appropriate to the outcome/exposure under study; final denominators are reported in the results.

### 2.6 Statistical Analysis

Analyses were conducted in IBM SPSS Statistics (version 29, MacOS, IBM Corp., Armonk, NY, USA). Categorical summaries are presented as counts and percentages. Agreement between *Alternaria* assays (SPT, whole‐extract sIgE, rAlt a 1) at each age was summarised using overall, positive and negative percentage agreement, Cohen's *κ* and McNemar's test for paired proportions. Kappa values were interpreted using conventional benchmarks: < 0.20 poor, 0.21–0.40 fair, 0.41–0.60 moderate, 0.61–0.80 substantial and > 0.80 almost perfect agreement.

Associations between *Alternaria* trajectories and disease trajectories were examined using Pearson's *χ* ^2^ with Cramér's *V* as effect size, and a linear‐by‐linear association test to evaluate trend across ordered categories (Never → Any → Persistent).

For dichotomous endpoints, we estimated relative risks (RRs) and 95% CIs using generalised linear models with Poisson distribution and log link, with robust (sandwich) standard errors [31]. Separate models were fitted for: asthma at 26; rhinitis at 26; persistent asthma (10–18–26); and persistent rhinitis (10–18–26). The exposure was the three‐level *Alternaria* trajectory (reference: Never); sex was included as an a priori covariate in all models. Model adequacy was summarised by the Akaike Information Criterion (AIC).

For continuous mechanistic outcomes, FeNO, spirometry and BHR were modelled as functions of *Alternaria* trajectories. Ln‐transformed FeNO at 18 and 26 years was analysed using univariate general linear models (UNIANOVA) with *Alternaria* trajectory as an ordered factor and sex as a covariate. Estimated marginal means and 95% CIs for each trajectory group were obtained and back‐transformed to yield sex‐adjusted geometric mean FeNO (ppb). Linear trend across trajectories (Never → Any → Persistent) was assessed using polynomial contrasts on the ordered trajectory variable.

Spirometric indices (FEV_1_% predicted, FVC% predicted and FEV_1_/FVC ratio at 10, 18 and 26 years) and log‐transformed dose–response slope (log[DRS^2^]) at 10 and 18 years were analysed similarly using UNIANOVA models with each spirometric measure as the dependent variable, *Alternaria* trajectory as an ordered factor and sex as a covariate. For each age and index, sex‐adjusted estimated marginal means, 95% CIs and a *p*‐value for linear trend across trajectories were reported. For visualisation of longitudinal patterns, repeated‐measures general linear models with age as the within‐subject factor and *Alternaria* trajectory plus sex as between‐subject factors were used to generate profile plots of sex‐adjusted means for FeNO, spirometric indices and log[DRS^2^].

All tests were two‐sided with *α* = 0.05. No formal multiplicity adjustment was applied; inference prioritised effect sizes and confidence intervals alongside *p*‐values. Reporting follows the Strengthening the Reporting of Observational Studies in Epidemiology (STROBE) recommendations [32].

## 3 Results

### 3.1 Prevalence of *Alternaria* Sensitisation and Allergic Outcomes Across the Life Course

*Alternaria* SPT sensitisation in this cohort was uncommon in early childhood (4.2% at 4 years) but increased by early adulthood (8.6% at 26 years). Whole‐extract *Alternaria* sIgE and rAlt a 1 showed similar age‐related increases (Figure 1, Table S1). Background atopy (any positive SPT) rose from 19.6% at 4 years to 45.7% at 26 years, and rhinitis from 5.4% to 42.1% over the same period, whereas asthma prevalence remained around 15% at most ages with only a modest peak in late adolescence (Figure 1, Table S1). For context, age‐specific sensitisation to other aeroallergens (e.g., HDM, grass/tree pollen, cat/dog, other moulds) and selected food allergens (e.g., milk, egg, peanut) is summarised in Tables S2 and S3. Co‐sensitisation patterns among *Alternaria*‐sensitised participants are shown in Tables S4 and S5.

**FIGURE 1:**
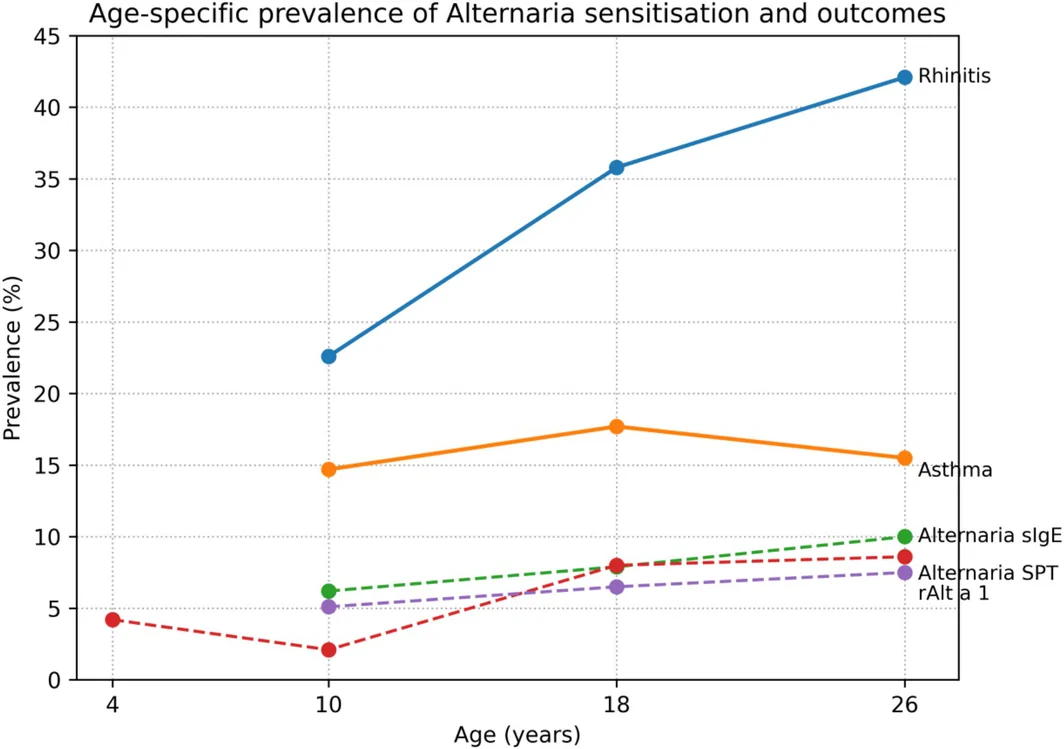
Prevalence of *Alternaria* measures and outcomes across ages (SPT, whole‐extract sIgE, rAlt a 1, ANY Alternaria, asthma, rhinitis). Age‐specific prevalence of *Alternaria* sensitisation by SPT, whole‐extract sIgE and rAlt a 1, overall atopy (≥ 1 positive SPT), asthma and rhinitis at 4, 10, 18 and 26 years. SPT ≥ 3 mm; sIgE ≥ 0.35 kUA/L. SPT, skin‐prick test; sIgE, serum specific IgE.

Preschool *Alternaria* SPT sensitisation was also examined prospectively and was associated with subsequent asthma, rhinitis, and bronchial hyper‐responsiveness across follow‐up (Table S6).

Participants contributing *Alternaria* measurements at 10, 18, and 26 years were broadly similar to those without measurements on key characteristics, with only small differences (Table S7).

### 3.2 Natural History of *Alternaria* Sensitisation

Longitudinal transitions in any *Alternaria* sensitisation (positive SPT and/or whole‐extract sIgE and/or rAlt a 1) are summarised in Figure 2. Between 4 and 10 years, sensitisation was relatively labile, with only about half of 4‐year positives remaining sensitised. Stability increased from 10 to 18 years, and by 18–26 years most sensitised individuals (over 80%) remained positive with very little de novo onset. Over the 10–26‐year span, around two‐thirds of those sensitised at 10 years were again sensitised at 26 years, and most 26‐year positives reflected persistence from late adolescence.

**FIGURE 2:**
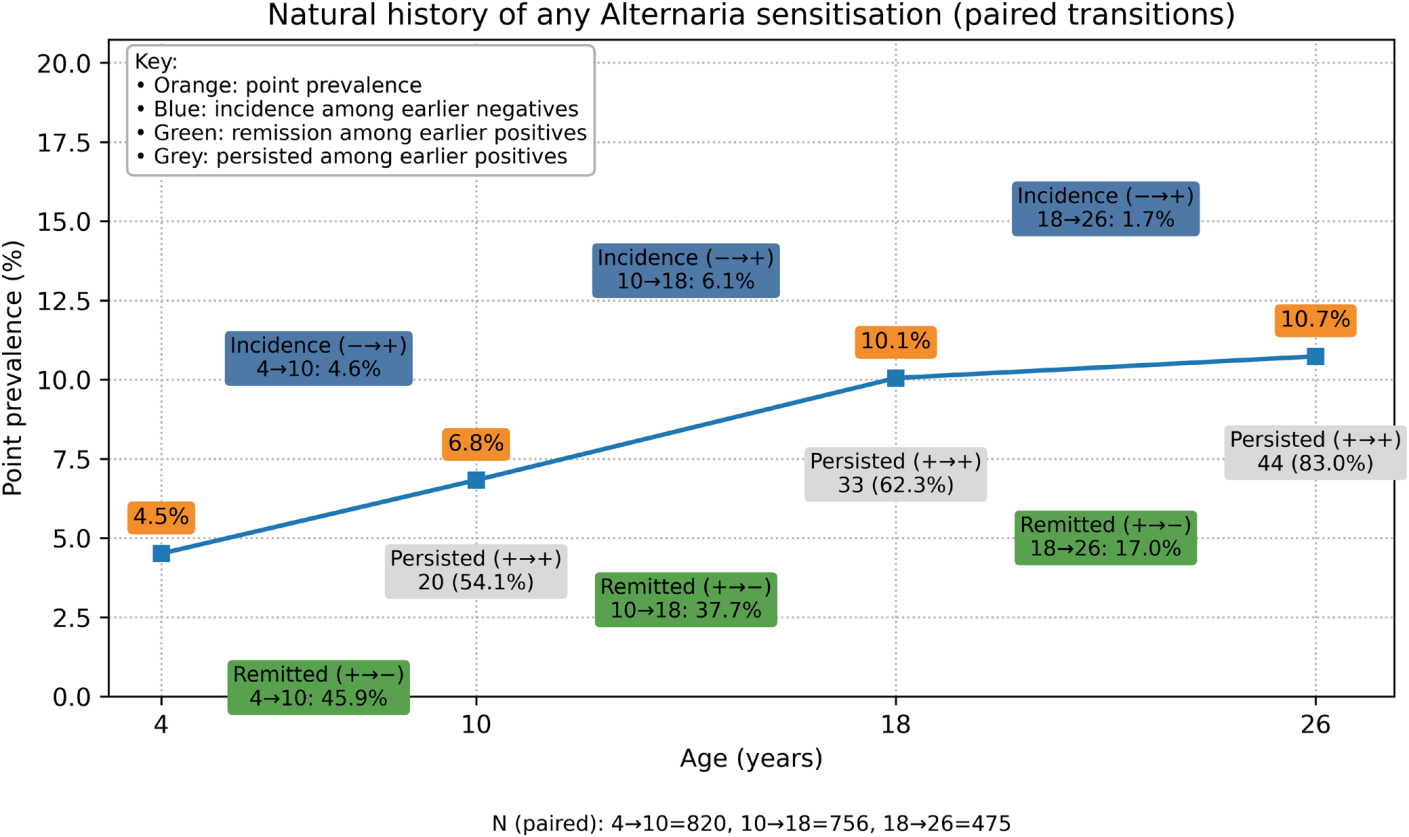
Natural history of any *Alternaria* sensitisation from ages 4–26 years. Orange boxes show point prevalence at each age. Blue boxes show incidence among earlier negatives (− → +) for each interval; green boxes show remission among earlier positives (+ → −); grey boxes show persistence among earlier positives (+ → +). Estimates use paired data (participants measured at both ages). Any *Alternaria* defined as SPT and/or whole‐extract sIgE ≥ 0.35 kUA/L and/or rAlt a 1 ≥ 0.35; at age 4 years only SPT was available.

### 3.3 Agreement Across *Alternaria* Assays (SPT, Whole‐Extract sIgE, rAlt a 1)

Agreement between assays strengthened with age and was substantial to almost perfect by late adolescence (Table S8). At 10 years, SPT showed only fair concordance with serum assays (agreement ~93%–95%, *κ* ≈ 0.17–0.20) and tended to be negative when serum was positive, whereas the two serum assays already aligned closely (agreement ~99%, *κ* ≈ 0.90) with whole‐extract sIgE slightly more often positive than rAlt a 1. By 18 years, SPT had largely converged with serum measures (agreement ~95%–97%, *κ* ≈ 0.66–0.74) and discordance was predominantly symmetrical, while concordance between the two serum assays remained very high. By 26 years, all pairings were near‐equivalent, with > 97% overall agreement, κ in the 0.87–0.90 range, and absolute discordance ≤ ~2%–3% for any comparison.

Among participants sensitised at baseline, higher *Alternaria* sIgE levels at 10 years predicted persistence of sensitisation at 18 years for both whole‐extract and rAlt a 1. At 18 years, rAlt a 1 levels showed weaker evidence of predicting persistence at 26 years, and whole‐extract 18 → 26 analyses were limited by sparse 26‐year whole‐extract sIgE data (Table S9).

### 3.4 Marker‐Wise Associations (Whole‐Extract sIgE vs. rAlt a 1)

Across ages, both whole‐extract *Alternaria* sIgE and rAlt a 1 were positively associated with asthma and rhinitis, but their relative performance varied by age (Table 1).

**TABLE 1:** Marker‐wise association with asthma and rhinitis at each age: whole‐extract *Alternaria* sIgE versus rAlt a 1.

Age	Marker	RR (95% CI)	*p*	AIC	*N*
*Asthma (sex‐adjusted Poisson; RR vs. marker‐negative)*					
10	rAlt a 1	3.01 (1.95–4.64)	< 0.001	695.2	899
10	Whole‐extract sIgE	2.44 (1.55–3.83)	< 0.001	699.1	899
18	rAlt a 1	3.39 (2.26–5.07)	< 0.001	493.5	555
18	Whole‐extract sIgE	2.93 (1.94–4.43)	< 0.001	496.0	555
26	rAlt a 1	2.13 (1.24–3.67)	0.006	425.5	478
26	Whole‐extract sIgE	2.52 (1.61–3.97)	< 0.001	423.1	478
*Rhinitis (sex‐adjusted Poisson; RR vs. marker‐negative)*					
10	rAlt a 1	2.57 (1.96–3.38)	< 0.001	1055.2	901
10	Whole‐extract sIgE	2.27 (1.71–3.00)	< 0.001	1058.1	901
18	Whole‐extract sIgE	2.39 (1.93–2.95)	< 0.001	784.8	555
18	rAlt a 1	2.34 (1.86–2.93)	< 0.001	788.2	555
26	rAlt a 1	1.52 (1.18–1.97)	0.001	778.1	479
26	Whole‐extract sIgE	1.54 (1.24–1.93)	< 0.001	777.9	479

*Note:* Marker‐wise associations of *Alternaria* biomarkers with asthma and rhinitis at each age. Sex‐adjusted Poisson models with log link and robust errors; RRs compare marker‐positive vs. marker‐negative. Lower AIC indicates better within‐age fit. *N* = complete cases for that age (26‐y models use matched samples). *p* values are Wald tests.

For asthma, rAlt a 1 consistently showed somewhat stronger associations and better model fit than whole‐extract in childhood and adolescence (e.g., at 10 years RR 3.01 vs. 2.44, with a lower AIC), indicating modestly better discrimination earlier in life. By 26 years, in an identical analytic sample, both markers remained significantly associated with asthma and had similar effect sizes, with whole‐extract only marginally stronger and slightly better fitting.

For rhinitis, the two assays performed very similarly at each age. Small differences in RRs and AICs alternated between whole‐extract and rAlt a 1 at 10 and 18 years, and by 26 years their effect sizes and model fit were essentially indistinguishable.

Given the high concordance between markers, subsequent trajectory analyses used ‘Any *Alternaria’* (positive by SPT and/or whole‐extract sIgE and/or rAlt a 1) as the sensitisation definition.

To contextualise these findings within broader atopy and co‐sensitisation, we compared participants sensitised to *Alternaria* with those sensitised to other allergens but not *Alternaria* at the same age; results were broadly comparable, with the main exception of a higher risk of rhinitis at 18 years (Table S10).

### 3.5 *Alternaria* Trajectories and Disease Trajectories (Three‐Group Schema)

Among participants with *Alternaria* measured at all three ages (*n* = 434), trajectory membership was: Never 84.1%, Any positive (non‐persistent) 11.1%, and Persistent 4.8%. In those with complete outcome data (*n* = 932 for each disease), asthma trajectories were Never 74%, Any positive 18.9% and Persistent 7.1%, whereas rhinitis trajectories were Never 39.2%, Any positive 50.5% and Persistent 10.3% (Table 2).

**TABLE 2:** *Alternaria* sensitisation trajectories (3‐group) versus asthma and rhinitis trajectories (3‐group).

*Alternaria* trajectory	Asthma: never	Asthma: any positive (non‐persistent)	Asthma: persistent	Total	Association
Never (negative all three timepoints)	278 (76.4%)	62 (17.0%)	24 (6.6%)	364	
Any positive (≥ 1 age), not persistent	26 (55.3%)	13 (27.7%)	8 (17.0%)	47	
Persistent (10, 18, 26)	11 (52.4%)	5 (23.8%)	5 (23.8%)	21	
Total	315	80	37	432	*χ* ^2^(4) = 17.95; *p* = 0.001; Cramér's *V* = 0.144; linear‐by‐linear trend *p* < 0.001
***Alternaria* trajectory**	**Rhinitis: Never**	**Rhinitis: Any positive (non‐persistent)**	**Rhinitis: Persistent**	**Total**	
Never (negative all three timepoints)	147 (40.3%)	180 (49.3%)	38 (10.4%)	365	
Any positive (≥ 1 age), not persistent	2 (4.2%)	29 (60.4%)	17 (35.4%)	48	
Persistent (10, 18, 26)	2 (9.5%)	11 (52.4%)	8 (38.1%)	21	
Total	151	220	63	434	*χ* ^2^(4) = 47.72; *p* < 0.001; Cramér's *V* = 0.234; linear‐by‐linear trend *p* < 0.001

*Note:* Row percentages within *Alternaria* trajectory (Never; Any positive ≥ 1 age, non‐persistent; Persistent at 10/18/26). Complete‐case denominators: Asthma *n* = 432, rhinitis *n* = 434. Associations: Asthma *χ* ^2^(4) = 17.95, *p* = 0.001, Cramér's *V* = 0.144; rhinitis *χ* ^2^(4) = 47.72, *p* < 0.001, *V* = 0.234. Linear‐by‐linear trend (df = 1) tests monotonic dose–response (Never → Any → Persistent vs. disease Never → Any → Persistent): *P* < 0.001 for both.

*Alternaria* trajectory was associated with both asthma and rhinitis trajectories (Table 2).

For asthma, there was a significant overall association (*χ* ^2^(4) = 17.95, *p* = 0.001; Cramér's *V* = 0.144) and a positive linear‐by‐linear trend, indicating that as *Alternaria* sensitisation progressed from Never to Any to Persistent, the distribution of asthma shifted towards more persistent disease (trend *p* < 0.001). Specifically, persistent asthma occurred in 6.6% of the Never group, 17% of the Any group and 23.8% of the Persistent group.

For rhinitis, the association was stronger (*χ* ^2^(4) = 47.72, *p* < 0.001; Cramér's *V* = 0.234) with a similarly positive linear‐by‐linear trend (*p* < 0.001): persistent rhinitis occurred in 10.4% of the Never group, 35.4% of the Any group and 38.1% of the Persistent group. Overall, Alternaria trajectories showed a modest gradient for asthma but a more pronounced gradient for rhinitis.

### 3.6 Associations Between Alternaria Sensitisation and Respiratory Outcomes

In sex‐adjusted Poisson models with robust standard errors (complete‐case *n* = 432–434), *Alternaria* sensitisation was associated with higher risks of both life‐course and age‐26 outcomes compared with the Never trajectory (Table 3). For 10/18/26 years outcomes, persistent *Alternaria* was associated with persistent asthma (RR 3.59, 95% CI 1.50–8.58) and persistent rhinitis (RR 3.65, 1.96–6.81). The Any positive (non‐persistent) trajectory also showed elevated risk for persistent asthma (RR 2.58, 1.23–5.41) and persistent rhinitis (RR 3.40, 2.09–5.53).

**TABLE 3:** Sex‐adjusted risk ratios for asthma and rhinitis outcomes by *Alternaria* sensitisation trajectory (3‐group).

Outcome	Any positive (≥ 1 age, non‐persistent)	*p*	Persistent (10, 18, 26)	*p*
Persistent asthma (10/18/26)	2.58 (1.23–5.41)	0.012	3.59 (1.50–8.58)	0.004
Persistent rhinitis (10/18/26)	3.40 (2.09–5.53)	< 0.001	3.65 (1.96–6.81)	< 0.001
Asthma at 26	2.28 (1.40–3.72)	0.001	2.76 (1.50–5.08)	0.001
Rhinitis at 26	1.61 (1.31–1.98)	0.001	1.37 (0.96–1.95)	0.082

*Note:* Poisson regression with log link and robust standard errors, adjusted for sex; reference = Never *Alternaria* trajectory. *p*‐values are Wald tests for each indicator. Denominators reflect complete‐case analyses. Ns vary (432–434) because models use complete cases for the relevant outcome among participants with *Alternaria* measured at 10/18/26.

For age‐26 outcomes, asthma risk at 26 years was increased in both the Any positive group (RR 2.28, 1.40–3.72) and the Persistent *Alternaria* group (RR 2.76, 1.50–5.08). For rhinitis at 26 years, risk remained clearly elevated in the Any positive group (RR 1.61, 1.31–1.98), whereas the Persistent group showed a similar direction of effect but with wider confidence intervals (RR 1.37, 0.96–1.95). In sensitivity analyses, additional adjustment for HDM sensitisation produced similar estimates, whereas further adjustment for polysensitisation burden attenuated effect sizes (Table S11).

At 18 years, sex‐adjusted geometric mean FeNO increased across *Alternaria* trajectories from 17.7 ppb in never‐sensitised participants to 24.0 ppb in those ever sensitised and 26.2 ppb in the persistent trajectory (p for trend = 0.022). At 26 years, the gradient was more marked (15.7, 21.4 and 26.2 ppb, respectively; p for trend < 0.001), corresponding to roughly 1.4–1.7‐fold higher FeNO in persistently sensitised individuals compared with the never‐sensitised trajectory (Figure 3, Table S12).

**FIGURE 3:**
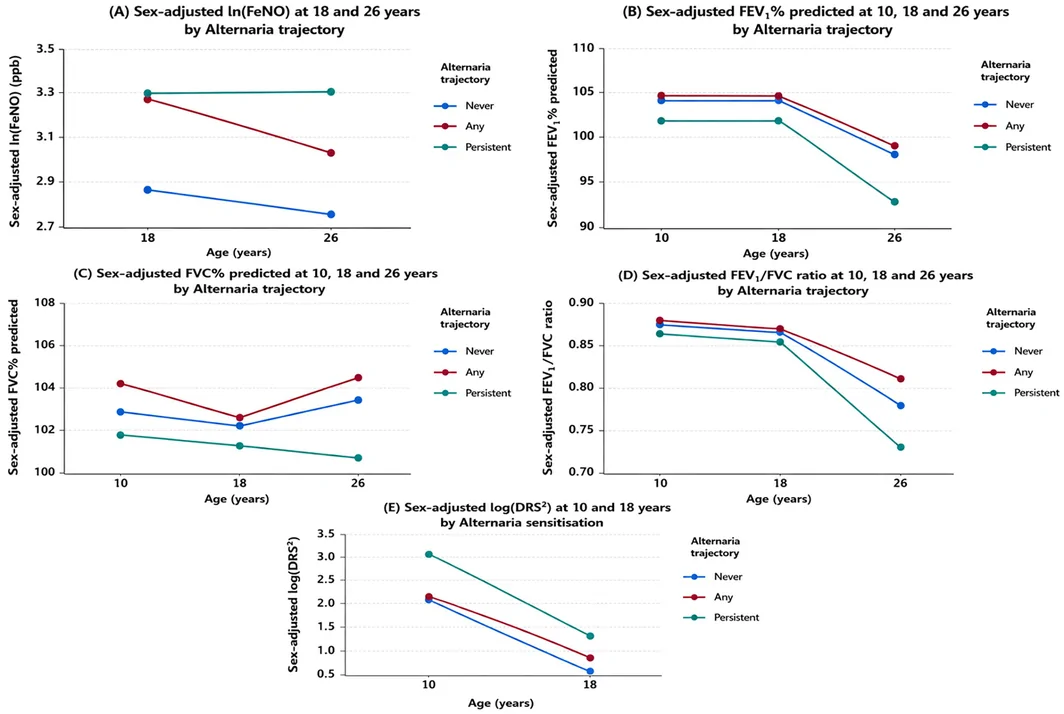
FeNO, spirometry and bronchial hyper‐responsiveness by Alternaria sensitisation trajectory. (A) Sex‐adjusted ln(FeNO) at 18 and 26 years. (B) FEV_1_% predicted, (C) FVC% predicted and (D) FEV_1_/FVC ratio at 10, 18 and 26 years. (E) Sex‐adjusted log‐transformed dose‐response slope (log[DRS^2^]) at 10 and 18 years. Values are estimated marginal means from general linear models with age and sex, stratified by Alternaria trajectory (Never, Any, Persistent).

In contrast, spirometric indices remained broadly normal and showed no convincing dose‐response by *Alternaria* trajectory. Mean FEV_1_% and FVC% predicted were close to or slightly above 100% at 10 and 18 years and around 100% at 26 years, with no clear linear trends (all *p* for trend ≥ 0.25). Similarly, FEV_1_/FVC showed no clear association with Alternaria trajectory at 10, 18 or 26 years (all p for trend ≥ 0.16; Figure 3, Table S13).

At 10 years, higher *Alternaria* trajectory was associated with greater BHR (overall *p* = 0.011; linear trend *p* = 0.003), with sex‐adjusted mean log(DRS^2^) of 2.12 (95% CI 1.93–2.31), 2.34 (1.86–2.81) and 3.22 (2.52–3.92) in the Never, Any and Persistent groups, respectively. At 18 years, the overall association was weaker (*p* = 0.081) but the linear trend remained significant (*p* = 0.032), with corresponding means of 0.60 (0.43–0.76), 0.81 (0.34–1.27) and 1.34 (0.68–2.00). Bronchial hyper‐responsiveness showed a similar pattern. Log‐transformed dose–response slope (log[DRS^2^]) decreased from 10 to 18 years in all *Alternaria* trajectories (*p* < 0.001 for the age effect), with no evidence that this change differed by trajectory (age×trajectory *p* = 0.57; Figure 2, Table S14).

## 4 Discussion

Using the IOWBC with harmonised assessments at ages 4, 10, 18 and 26 years, we show that *Alternaria* sensitisation emerges predominantly around late adolescence and tends to persist into early adulthood, and that the persistence of sensitisation is linked to the persistence of asthma and rhinitis, and to persistently higher FeNO levels and greater BHR. Among those with complete measurements across 10–18–26 years, 4.8% exhibited persistent *Alternaria* sensitisation, who bore a disproportionately higher burden of persistent asthma and rhinitis, with clear graded trends across “Never → Any (non‐persistent) → Persistent” trajectories. In cross‐sectional risk models, rAlt a 1 outperformed whole‐extract sIgE for asthma at 10 and 18 years, while at 26 years both assays performed similarly.

Because *Alternaria* sensitisation frequently co‐occurs with sensitisation to other aeroallergens, we undertook sensitivity analyses to explore potential confounding by concomitant atopy. Estimates were broadly similar after additional adjustment for HDM sensitisation, suggesting that the observed associations are not solely explained by HDM co‐sensitisation. By contrast, adjustment for polysensitisation burden attenuated associations, consistent with *Alternaria* sensitisation being closely linked to broader atopic propensity; this analysis is therefore best interpreted as a conservative atopic‐burden sensitivity analysis rather than a definitive assessment of independence.

Agreement between SPT, whole‐extract sIgE and rAlt a 1 was already substantial by 18 years and was near‐perfect by 26 years, supporting the convergence of diagnostic modalities later in the life course. To our knowledge, this is one of the first population‐based cohort analyses to (i) define *Alternaria* sensitisation trajectories spanning adolescence to adulthood and (ii) compare component‐resolved diagnostics (rAlt a 1) against whole‐extract sIgE at multiple ages within the same individuals. The trajectory design moves beyond single‐age snapshots to quantify the timing and persistence of sensitisation and its life‐course relation to asthma and rhinitis.

The clinical salience of *Alternaria* sensitisation is well documented: cross‐sectional surveys and time‐series studies report associations with asthma, acute exacerbations, emergency utilisation and even epidemic “thunderstorm” asthma events [1, 5, 6, 7]. Our findings in the IOWBC extend this literature by demonstrating that not merely *Alternaria* status at a single time point, but the trajectory of *Alternaria* sensitisation, tracks with the trajectory of asthma and rhinitis. This echoes earlier population work linking *Alternaria* to asthma risk [8, 9, 10] and more recent cohort evidence of downstream airway BHR among sensitised non‐asthmatics [12], but adds a life‐course, three‐time‐point perspective. The natural history of *Alternaria* allergy is of remission and relapse during childhood but it becomes stable once the child reaches the age of 18 years. *Alternaria* sensitisation was less common in childhood associated with lower concordance between skin test and IgE. It rises rapidly during adolescence and remains relatively high and persistent during adult life and exhibits high concordance between skin test, IgE to whole extract and Alt a1.

In European multicentre data (GA^2^LEN), SPT to inhalant allergens—including moulds—demonstrated clinical relevance across centres.^2^ Our agreement analyses show that, by late adolescence and early adulthood, SPT, whole‐extract sIgE and rAlt a 1 largely concur, supporting pragmatic interchangeability when a single test is feasible. At younger ages, however, rAlt a 1 provided stronger asthma risk signals than whole‐extract sIgE, consistent with Alt a 1 being the major *Alternaria* allergen and a marker of genuine sensitisation. This aligns with mechanistic immunochemistry and supports component‐resolved diagnostics as a useful first‐line assay when the clinical question is asthma‐focused in youth.

Longitudinal IOW work has previously highlighted adolescence as a critical period for the consolidation of respiratory phenotypes [16, 17, 18, 33]. We now demonstrate that *Alternaria* sensitisation follows a similar developmental pattern, with higher incident sensitisation and stronger persistence from 18 → 26 years than at earlier ages—information that is directly actionable for timing surveillance and counselling.

There was a strong association between Alternaria sensitisation and asthma and rhinitis. However, most Alternaria‐sensitised individuals were also sensitised to other allergens and polysensitisation was common; therefore, it is not possible to be certain of a causal role for Alternaria sensitisation in observational data. In sensitivity analyses, estimates were broadly similar after adjustment for HDM sensitisation, whereas further adjustment for polysensitisation burden attenuated associations. Nevertheless, given that Alternaria is a potent allergen and has been shown to induce allergic immune responses [34, 35], and is associated with asthma development and exacerbations, the observed associations are likely to be clinically relevant. Our trajectory analysis demonstrating strong associations between persistent Alternaria sensitisation and persistent asthma and rhinitis over the life course supports a contribution of Alternaria to respiratory allergic disease.

Our finding highlights three main messages. Adolescence is a period of change [36, 37]. In the IOWBC, we have observed an overall increase in the prevalence of asthma, rhinitis, *Alternaria* sensitisation and overall atopy. Incident *Alternaria* sensitisation clustered around 18 years, and once present, frequently persisted to 26. This may provide opportunities to assess those with incident asthma and rhinitis for *Alternaria* sensitisation takes measures to reduce exposure in the environment and optimisation of anti‐inflammatory therapy in sensitised adolescents/young adults, particularly ahead of late‐summer/early‐autumn spore peaks and during thunderstorm‐prone conditions [38, 39, 40]. It also calls for development of allergen immunotherapy products for *Alternaria*, especially during adolescence before a more persistent allergic trajectory sets in [20, 41].

Secondly, any *Alternaria* positivity matters; persistence matters more. Even non‐persistent “Any positive” trajectories carried elevated risks of asthma and rhinitis; persistent *Alternaria* showed the largest proportions of persistent disease. Clinically, any objective *Alternaria* signal should prompt a control review and an action plan for high‐risk weather/seasonal periods.

Thirdly, assay choice can be age‐tailored. In childhood/adolescence, rAlt a 1 is at least as informative—and often more informative—than whole‐extract sIgE for asthma risk; by 26 years, either assay is acceptable. Where only one test is feasible, rAlt a 1 is a reasonable first choice in younger patients with asthma‐focused questions; SPT or whole‐extract remain suitable alternatives, especially when component testing is unavailable.

The strengths of this study are multi‐fold, including a whole‐population birth cohort from a defined UK island community, repeated assessments with harmonised protocols, the availability of SPT, whole‐extract sIgE and rAlt a 1 across multiple ages, and a trajectory‐based analytic framework linking sensitisation to asthma/rhinitis courses.

As with other studies, this study has some limitations. These include attrition and missingness inherent to long‐term follow‐up, and some analyses required complete *Alternaria* measurements across ages 10, 18 and 26 years. Compared with the birth cohort, the complete‐case analytic sample included fewer males and a higher prevalence of rhinitis at age 26 years (Table S7). This may limit generalisability and could modestly influence the magnitude of associations, although the overall pattern of findings was consistent across analyses. Additional limitations include the low absolute prevalence of Alternaria sensitisation (wider CIs for certain estimates), and the absence of contemporaneous environmental exposure metrics (e.g., outdoor spore counts, indoor damp/mould) that could refine causal inference. Immunotherapy evidence in *Alternaria*‐sensitised patients remains limited and heterogeneous [19, 20], so our data are best viewed as risk‐stratification evidence rather than therapeutic guidance.

The IOW Birth Cohort is broadly representative of a UK general population birth cohort, but the findings may not generalise fully to settings with different climate, housing characteristics, fungal spore burden, ethnicity, or background atopic risk. In particular, *Alternaria* exposure and sensitisation patterns vary geographically and seasonally, and associations may differ in regions with higher mould exposure or in more ethnically diverse populations. Nonetheless, the life‐course trajectory approach and the consistent direction of associations across childhood to adulthood support the broader relevance of persistent *Alternaria* sensitisation as a marker of respiratory allergic disease risk.

Future work should move beyond characterising trajectories to identifying upstream determinants of the development and persistence of *Alternaria* sensitisation across the life course. Socioeconomic and housing factors are plausible contributors—through differences in indoor dampness/mould, ventilation and crowding—alongside other early‐life and environmental exposures [42, 43]. Analyses incorporating detailed sociodemographic and housing data could help clarify why some individuals transition to persistent sensitisation and, in turn, inform targeted prevention and exposure‐reduction strategies.

## 5 Conclusions

In the IOWBC, *Alternaria* sensitisation—particularly when persistent across adolescence into adulthood—is associated with more persistent asthma and rhinitis. rAlt a 1 provides stronger asthma risk discrimination in youth than whole extract sIgE, while both assays converge in adulthood as agreement between tests becomes high. These life‐course data refine the clinical relevance of *Alternaria* within a population cohort and identify adolescence as a key window for targeted risk communication and prevention.

## Author Contributions

S.H.A. conceptualised the study and discussed with R.K. and P.V. M.L. performed data analysis, guided by S.H.A. and H.Z. S.H.A. and M.L. wrote the first draft. The manuscript was reviewed by all authors and edited and revised accordingly. All authors have read and agreed to the final submitted draft.

## Funding

The authors have nothing to report.

## Conflicts of Interest

The authors declare no conflicts of interest.

## Supporting information


**Table S1:** Age‐specific prevalence of *Alternaria* sensitisation, atopy, asthma and rhinitis in the Isle of Wight Birth Cohort (ages 4, 10, 18 and 26 years).
**Table S2:** Sensitisation to common aeroallergens by age (SPT).
**Table S3:** Co‐sensitisation to other aeroallergens among Alternaria‐sensitised participants.
**Table S4:** Sensitisation to selected food allergens by age (SPT).
**Table S5:** Co‐sensitisation to selected food allergens among Alternaria‐sensitised participants (SPT).
**Table S6:** Preschool Alternaria sensitisation and subsequent asthma, rhinitis and bronchial hyperresponsiveness across follow‐up.
**Table S7:** Comparison of the complete‐case analytic sample with the birth cohort.
**Table S9:** Baseline Alternaria sIgE level predicts persistence of sensitisation at the subsequent follow‐up (10 → 18 and 18 → 26 years).
**Table S10:** Comparison of asthma and rhinitis risk in Alternaria‐sensitised participants versus non‐Alternaria atopy at each age.
**Table S11:** Sensitivity analyses for associations between Alternaria sensitisation trajectories (ages 10, 18 and 26 years) and asthma/rhinitis at ages 18 and 26.
**Table S12:** Fractional exhaled nitric oxide (FeNO) at 18 and 26 years by Alternaria sensitisation trajectory.
**Table S13:** Sex‐adjusted spirometry by Alternaria sensitisation trajectory.
**Table S14:** Sex‐adjusted log(DRS2) at 10 and 18 years by Alternaria sensitisation trajectory.

## Data Availability

The data that support the findings of this study are available from the corresponding author upon reasonable request.
