# Supplementary material for: Natural History of Alternaria Sensitisation and Association With Asthma and Rhinitis From Childhood to Adulthood
Source: Allergy. 2026 May 18;81(11):3936–46. doi: 10.1111/all.70394 (PMC13753657; doi:10.1111/all.70394)
Supplement: Supplementary file 1 [file ALL-81-3936-s001.docx]

### **Supplementary material**

## *Supplementary Methods*: Serum specific IgE measurement and sample handling

## At ages 10, 18 and 26 years, venous blood was collected during scheduled cohort follow-up visits. Immediately after collection of blood sample, serum was separated, aliquoted and stored frozen (≤−20°C) under controlled biobank conditions until analysis. Whole-Alternaria alternat extract and recombinant Alt a 1 allergen-specific IgE (sIgE) were quantified using the ImmunoCAP fluorescence enzyme immunoassay platform (Thermo Fisher Scientific/Phadia AB, Uppsala, Sweden) in accordance with the manufacturer’s instructions and calibration procedures, with results reported in kUA/L. Positivity was defined a priori as sIgE ≥0.35 kUA/L.

## To maintain sample integrity, serum was stored as aliquots to minimise freeze–thaw exposure; where repeat testing was required, unused aliquots were preferentially analysed. Published evidence supports the stability of IgE in frozen sera: total and allergen-specific IgE remain measurable after long-term storage at −20°C (including >30 years), and comparative CAP/UniCAP data show no evidence of IgE degradation over multi-year storage intervals.^1^ In addition, sIgE has been shown to be stable across common pre-analytical handling conditions, with minimal impact from a single freeze–thaw cycle, supporting the use of biobanked samples when freeze–thaw is minimised. Assays were performed with routine internal quality control and run validity checks (including manufacturer-provided controls and acceptance criteria) in line with laboratory standard operating procedures

### Supplementary table 1. Age-specific prevalence of *Alternaria* sensitisation, atopy, asthma and rhinitis in the Isle of Wight Birth Cohort (ages 4, 10, 18 and 26 years).

| Measure | 4 y | 10 y | 18 y | 26 y |
| --- | --- | --- | --- | --- |
| *Alternaria* SPT | 41/975 (4.2) | 22/1035 (2.1) | 68/851 (8.0) | 48/556 (8.6) |
| Any SPT | 192/978 (19.6) | 279/1036 (26.9) | 351/851 (41.2) | 254/556 (45.7) |
| *Alternaria* sIgE (whole) | – | 56/901 (6.2) | 44/556 (7.9) | 49/488 (10.0) |
| rAlt a 1 sIgE | – | 46/901 (5.1) | 36/556 (6.5) | 36/481 (7.5) |
| Inhalant screen (blood) | – | 314/952 (33.0) | 248/560 (44.3) | – |
| Asthma | 182/1214 (15.0) | 201/1368 (14.7) | 231/1305 (17.7) | 160/1030 (15.5) |
| Rhinitis | 66/1214 (5.4) | 308/1362 (22.6) | 468/1309 (35.8) | 435/1033 (42.1) |

Supplementary table 1. Values are n/N (%). *Alternaria* SPT: wheal ≥3 mm over negative control to *Alternaria* extract (ALK). Any SPT (atopy): ≥1 positive SPT to the standard inhalant panel (included grass and tree pollens, house dust mite, cat and dog dander, and common moulds (including *Alternaria*). *Alternaria* sIgE (whole) and rAlt a 1 sIgE: positivity defined as ≥0.35 kUA/L.  Denominators reflect available cases at each age; measures not assayed at specific ages are shown as “–”.

## Supplementary table 2. Sensitisation to common aeroallergens by age (SPT)

| Allergen | 4y | 10y | 18y | 26y |
| --- | --- | --- | --- | --- |
| HDM | 105/977 (10.7%) | 199/1035 (19.2%) | 244/851 (28.7%) | 180/556 (32.4%) |
| Grass | 69/976 (7.1%) | 134/1036 (12.9%) | 203/851 (23.9%) | 147/556 (26.4%) |
| Tree | — | 19/1035 (1.8%) | 49/851 (5.8%) | 29/556 (5.2%) |
| Cat | 51/976 (5.2%) | 82/1035 (7.9%) | 100/851 (11.8%) | 69/556 (12.4%) |
| Dog | 19/976 (1.9%) | 46/1035 (4.4%) | 90/851 (10.6%) | 49/556 (8.8%) |
| Cladosporium | 15/975 (1.5%) | 11/1035 (1.1%) | 25/851 (2.9%) | 6/556 (1.1%) |
| Aspergillus | — | — | — | 7/556 (1.3%) |
| Alternaria | 41/975 (4.2%) | 22/1035 (2.1%) | 68/851 (8.0%) | 48/556 (8.6%) |

Supplementary Table 2. Sensitisation to common aeroallergens by age (SPT). Values are n/N (%) with N denoting the number of participants with available skin prick testing (SPT) results for each allergen at the specified age. HDM denotes house dust mite. Tree pollen was not tested at age 4 years, and *Aspergillus* was tested at age 26 years only.

## Supplementary table 3. Co-sensitisation to other aeroallergens among Alternaria-sensitised participants

| Co-sensitisation | 4y | 10y | 18y | 26y |
| --- | --- | --- | --- | --- |
| Any other aeroallergen (≥1) | 23/41 (56.1%) | 17/22 (77.3%) | 58/68 (85.3%) | 40/48 (83.3%) |
| HDM | 17/41 (41.5%) | 8/22 (36.4%) | 37/68 (54.4%) | 27/48 (56.2%) |
| Grass | 13/41 (31.7%) | 16/22 (72.7%) | 48/68 (70.6%) | 36/48 (75.0%) |
| Tree | — | 5/22 (22.7%) | 16/68 (23.5%) | 7/48 (14.6%) |
| Cat | 8/41 (19.5%) | 3/22 (13.6%) | 26/68 (38.2%) | 20/48 (41.7%) |
| Dog | 4/41 (9.8%) | 3/22 (13.6%) | 26/68 (38.2%) | 17/48 (35.4%) |
| Cladosporium | 3/41 (7.3%) | 3/22 (13.6%) | 12/68 (17.6%) | 4/48 (8.3%) |
| Aspergillus | — | — | — | 3/48 (6.2%) |

Supplementary Table 3. Co-sensitisation to other aeroallergens among Alternaria-sensitised participants (SPT). Values are n/N (%), where N is the number of participants who were *Alternaria* SPT-positive at the specified age. “Any other aeroallergen” denotes sensitisation to ≥1 of the other aeroallergens tested at that age (excluding Alternaria). HDM denotes house dust mite. Tree pollen was not tested at age 4 years, and *Aspergillus* was tested at age 26 years only.

## Table Supplementary Table 4. Sensitisation to selected food allergens by age (SPT)

| Allergen | 4y | 10y | 18y | 26y |
| --- | --- | --- | --- | --- |
| Milk | 11/977 (1.1%) | 3/1034 (0.3%) | 9/851 (1.1%) | 0/556 (0.0%) |
| Egg | 7/977 (0.7%) | 4/1034 (0.4%) | 8/851 (0.9%) | 2/556 (0.4%) |
| Peanut | 10/975 (1.0%) | 18/1034 (1.7%) | 54/851 (6.3%) | 21/556 (3.8%) |
| Soya | 2/974 (0.2%) | 2/1034 (0.2%) | 6/851 (0.7%) | 1/556 (0.2%) |
| Cod | 5/939 (0.5%) | 8/1034 (0.8%) | 15/851 (1.8%) | 3/556 (0.5%) |
| Wheat | 1/977 (0.1%) | 24/1034 (2.3%) | 168/851 (19.7%) | — |
| Wheat flour | — | — | — | 2/556 (0.4%) |

## Supplementary Table 4. Sensitisation to selected food allergens by age (SPT). Values are n/N (%) with N denoting the number of participants with available skin prick testing (SPT) results for each allergen at the specified age. Wheat extract was tested at ages 4, 10 and 18 years, while wheat flour was tested at age 26 years only; these are therefore reported separately.

## Table Supplementary Table 5. Co-sensitisation to selected food allergens among *Alternaria*-sensitised participants

| Food co-sensitisation | 4y | 10y | 18y | 26y |
| --- | --- | --- | --- | --- |
| Milk | 6/41 (14.6%) | 3/22 (13.6%) | 6/68 (8.8%) | 0/48 (0.0%) |
| Egg | 2/41 (4.9%) | 0/22 (0.0%) | 4/68 (5.9%) | 1/48 (2.1%) |
| Peanut | 1/41 (2.4%) | 2/22 (9.1%) | 12/68 (17.6%) | 4/48 (8.3%) |
| Soya | 1/41 (2.4%) | 0/22 (0.0%) | 2/68 (2.9%) | 0/48 (0.0%) |
| Cod | 1/38 (2.6%) | 2/22 (9.1%) | 6/68 (8.8%) | 1/48 (2.1%) |
| Wheat | 0/41 (0.0%) | 2/22 (9.1%) | 41/68 (60.3%) | — |
| Wheat flour | — | — | — | 0/48 (0.0%) |

## Supplementary Table 5. Co-sensitisation to selected food allergens among Alternaria-sensitised participants (SPT).Values are n/N (%), where N is the number of participants who were *Alternaria* SPT-positive at the specified age and had SPT results available for the specified food allergen. Wheat extract was tested at ages 4, 10 and 18 years, while wheat flour was tested at age 26 years only; these are therefore reported separately.

## Supplementary Table 6. Preschool *Alternaria* sensitisation and subsequent asthma, rhinitis and bronchial hyperresponsiveness across follow-up.

| Table 6 A: Preschool age *Alternaria* sensitisation and development of asthma and rhinitis and BHR until adult life. | | | | |
| --- | --- | --- | --- | --- |
|  | *Alternaria* sensitised at 4 years | | 95% CI | P= |
|  | Yes | No |  |  |
| Age 4 | | | | |
| Asthma | 34.1% (14/41) | 15.6% (146/788) | 2.80 (1.43-5.47) | .004 |
| Rhinitis | 17.1% (7/41) | 5.7% (53/934) | 3.42 (1.45-8.08) | .01 |
| Age 10 | | | | |
| Asthma | 36.8% (14/38) | 14.4% (129/898) | 3.48 (1.80-6.90) | <.001 |
| New onset asthma | 13.2% (5/38) | 6.2% (56/898) |  | 0.01 |
| Rhinitis | 50% (19/38) | 22.9% (205/895) | 3.37 (1.75-6.48) | <.001 |
| BHR +ve* | 56.7% (17/30) | 28.5% (167/586) | 1.99 (1.42-2.79) | .002 |
| Age 18 | | | | |
| Asthma | 37.8% (14/37) | 16.5% (142/859) | 3.07 (1.54-6.12) | .002 |
| New onset asthma | 17.1% (6/35) | 9.1% (74/811) |  | 0.01 |
| Rhinitis | 78.4% (29/37) | 35.3% (305/865) | 6.66 (3.01-14.74) | <.001 |
| BHR +ve | 23.5% (4 /17) | 6.2% (29/466) | 3.78 (1.50-9.55) | .02 |
| Age 26 | | | | |
| Asthma | 29.4% (10/34) | 13.4% (92/688) | 2.70 (1.25-5.83) | .014 |
| New onset asthma | 3.0% (1/33) | 3.2% (21/665) |  | - |
| Rhinitis | 64.7% (22/34) | 41.7% (288/691) | 2.57 (1.25-5.27) | .007 |
| *defined as PC_20_ Methacholine <8mg/ml) | | | | |

| Table 6 B: Preschool age *Alternaria* sensitisation and association with lung function. | | | | |
| --- | --- | --- | --- | --- |
|  | *Alternaria* sensitised at 4 years (mean) | | Mean Difference (SD) | P= |
|  | Yes | No |  |  |
| Age 10 | | | | |
| FEV_1_ | 2.02L | 2.03L | -.005 (.06) | .47 |
| FEV_1_/FVC ratio | 0.86 | 0.88 | -.014 (.15) | .17 |
| FEF_25-75_ | 2.31L | 2.36L | -.051 (.13) | .35 |
| BHR (DRS) | -194.34 | -55.18 | -139.16 (30.38) | <.001 |
| Age 18 | | | | |
| FEV_1_ | 3.77L | 4.01L | -.245 (.13) | .04 |
| FEV_1_/FVC ratio | 0.84L | 0.88 | -.036 (.02) | .05 |
| FEF_25-75_ | 3.85 | 4.47L | -.622 (.20) | .002 |
| BHR (DRS) | -52.50 | -12.10 | -40.40 (17.79) | .01 |
| Age 26 | | | | |
| FEV_1_ | 3.57L | 3.87L | -.305 (.18) | .05 |
| FEV_1_/FVC ratio | 0.78L | 0.79 | -.008 (.03) | .39 |
| FEF_25-75_ | 3.46L | 3.83L | -.371 (.28) | .10 |

Supplementary Table 6. *Alternaria* sensitisation at 4 years was defined by skin prick testing (SPT) positivity. Associations with later outcomes at ages 10, 18 and 26 years are presented as risk ratios (RRs) with 95% confidence intervals from Poisson regression with robust standard errors (sex-adjusted). Bronchial hyperresponsiveness (BHR) was defined as PC20 <8 mg/mL; dose–response slope (DRS) is reported as a continuous marker of BHR severity. Spirometry indices are presented as adjusted mean differences (with 95% CIs) comparing Alternaria-sensitised versus non-sensitised participants. Denominators vary by outcome due to complete-case availability at each age.

Supplementary Table 7. Comparison of the complete-case analytic sample with the birth cohort

| Characteristic | Analytic sample n/N (%) | Not in sample n/N (%) | p-value | Std diff |
| --- | --- | --- | --- | --- |
| Male sex | 112/255 (43.9%) | 674/1281 (52.6%) | 0.011 | -0.17 |
| Atopy at 4y | 37/222 (16.7%) | 155/758 (20.4%) | 0.212 | -0.10 |
| Atopy at 10y | 67/255 (26.3%) | 212/781 (27.1%) | 0.786 | -0.02 |
| Atopy at 18y | 110/255 (43.1%) | 241/596 (40.4%) | 0.463 | 0.05 |
| Atopy at 26y | 124/255 (48.6%) | 130/301 (43.2%) | 0.200 | 0.11 |
| Asthma at 10y | 28/255 (11.0%) | 173/1113 (15.5%) | 0.063 | -0.13 |
| Rhinitis at 10y | 67/255 (26.3%) | 241/1107 (21.8%) | 0.121 | 0.11 |
| Asthma at 18y | 38/255 (14.9%) | 193/1050 (18.4%) | 0.192 | -0.09 |
| Rhinitis at 18y | 96/255 (37.6%) | 372/1054 (35.3%) | 0.482 | 0.05 |
| Asthma at 26y | 35/255 (13.7%) | 125/775 (16.1%) | 0.358 | -0.07 |
| Rhinitis at 26y | 130/255 (51.0%) | 305/778 (39.2%) | <0.001 | 0.24 |

At age 4 years, only SPT was available; specific IgE and rAlt a 1 were not measured.

Supplementary Table 7. The complete-case analytic sample was defined as participants with non-missing Alternaria trajectory status. Birth cohort N=1536; analytic sample n=255; not in analytic sample n=1281. Values are n/N (%), where N may vary by characteristic due to missingness. p-values are from χ² tests (or Fisher’s exact where appropriate). Std diff = standardised difference for proportions.

Supplementary table 8. Agreement among *Alternaria* measures (paired complete cases)

| Age | Pair | N | Overall agreement | κ | McNemar p |
| --- | --- | --- | --- | --- | --- |
| 10 | SPT ↔ sIgE | 898 | 93.4% | 0.168 | <0.001 |
| 10 | SPT ↔ rAlt a 1 | 898 | 94.6% | 0.200 | <0.001 |
| 10 | IgE ↔ rAlt a 1 | 901 | 98.9% | 0.896 | 0.002 |
| 18 | SPT ↔ sIgE | 533 | 95.3% | 0.658 | 0.690 |
| 18 | SPT ↔ rAlt a 1 | 533 | 96.8% | 0.744 | 0.332 |
| 18 | sIgE ↔ rAlt a 1 | 556 | 98.6% | 0.892 | 0.008 |
| 26 | SPT ↔ IgE | 483 | 97.7% | 0.869 | 0.227 |
| 26 | SPT ↔ rAlt a 1 | 476 | 98.3% | 0.883 | 0.727 |
| 26 | IgE ↔ rAlt a 1 | 481 | 98.5% | 0.904 | 0.016 |

Supplementary table 8. Overall agreement is % concordant results; κ is Cohen’s kappa (chance-corrected agreement); McNemar *p* tests asymmetry of discordant pairs; *N* is the number with both tests at that age. SPT = skin-prick test (wheal ≥3 mm vs negative control); sIgE = whole-extract *Alternaria* sIgE ≥0.35 kUA/L; rAlt a 1 = recombinant Alt a 1 sIgE ≥0.35 kUA/L.

## Supplementary Table 9. Baseline *Alternaria* sIgE level predicts persistence of sensitisation at the subsequent follow-up (10→18 and 18→26 years).

| Assay / window | Baseline-positive N | Persistent n (%) | Temporary n (%) | Baseline IgE median (IQR), temporary | Baseline IgE median (IQR), persistent | RR per 1-unit ln(IgE) (95% CI) | p |
| --- | --- | --- | --- | --- | --- | --- | --- |
| Whole extract 10→18 | 24 | 18 (75.0) | 6 (25.0) | 0.59 (0.36–0.76) | 5.91 (1.77–18.23) | 1.27 (1.08–1.49) | 0.003 |
| Alt a 1 10→18 | 23 | 18 (78.3) | 5 (21.7) | 0.77 (0.39–1.15) | 10.09 (1.67–30.58) | 1.21 (1.05–1.38) | 0.007 |
| Alt a 1 18→26 | 20 | 16 (80.0) | 4 (20.0) | 1.96 (0.94–10.22) | 6.70 (1.73–19.53) | 1.09 (0.91–1.31) | 0.355 |

## Supplementary Table 9. Whole-extract and rAlt a 1 sIgE levels at baseline and persistence of *Alternaria* sensitisation at the next time-point. Analyses were restricted to participants sensitised at baseline (sIgE ≥0.35 kUA/L). “Persistent” indicates sensitisation at the subsequent follow-up; “temporary” indicates loss of sensitisation. Baseline sIgE is summarised as median (IQR). Risk ratios (RR) are sex-adjusted Poisson models with robust SEs per 1-unit increase in ln(sIgE). Whole-extract 18→26 modelling was not undertaken due to limited availability of 26-year whole-extract sIgE data.

Supplementary table 10. Comparison of asthma and rhinitis risk in Alternaria-sensitised participants versus non-*Alternaria* atopy at each age.

|  | *Alternaria* sensitised at: | Atopic, but not sensitised to *Alternaria* at: |  |  |
| --- | --- | --- | --- | --- |
| Age 4 | Yes at age 4 | No at age 4 | 95% CI | p |
| Asthma | 34.1% (14/41) | 37.1% (56/151) | 0.88 (0.43-1.82) | .44 |
| Rhinitis | 17.1% (7/41) | 16.6% (25/151) | 1.04 (0.41-2.60) | .55 |
| Age 10 | Yes at age 10 | No at age 10 | 95% CI | P |
| Asthma | 31.8% (7/22) | 36.2% (93/257) | 0.82 (0.32-2.09) | .44 |
| Rhinitis | 63.3% (14/22) | 48.2% (124/257) | 1.88 (0.76-4.63) | .12 |
| Age 18 | Yes at age 18 | No at age 18 | 95% CI | P |
| Asthma | 36.8% (25/68) | 31.6% (89/282) | 1.26 (0.73-2.19) | 0.25 |
| Rhinitis | 76.5% (52/68) | 64.0 (181/283) | 1.83 (1.00-3.37) | 0.03 |
| Age 26 | Yes at age 26 | No at age 26 |  |  |
| Asthma | 33.3% (16/48) | 26.8% (55/205) | 1.36 (0.69-2.68) | .23 |
| Rhinitis | 68.8% (33/48) | 65.0% (134/206) | 1.18 (0.60-2.32) | .38 |

Supplementary Table 10. Comparison of asthma and allergic rhinitis in participants sensitised to *Alternaria* versus those sensitised to other allergens (atopic) but not *Alternaria* at the same age. Values are percentages with counts (n/N) for each outcome. The comparison group comprises participants with skin prick test evidence of sensitisation to ≥1 allergen other than *Alternaria* at the corresponding age. Effect estimates are presented as risk ratios with 95% confidence intervals (p values shown).

Supplementary Table 11. Sensitivity analyses for associations between *Alternaria* sensitisation trajectories (ages 10, 18 and 26 years) and asthma/rhinitis at ages 18 and 26.

| Outcome (age) | Model | N | Any vs Never, RR (95% CI) | p (Any) | Persistent vs Never, RR (95% CI) | p (Persistent) |
| --- | --- | --- | --- | --- | --- | --- |
| Asthma (18y) | Sex | 255 | 1.77 (0.79–3.97) | 0.165 | 3.27 (1.69–6.34) | <0.001 |
| Asthma (18y) | Sex + HDM | 255 | 1.51 (0.70–3.27) | 0.292 | 2.16 (0.97–4.80) | 0.060 |
| Asthma (18y) | Sex + polysensitisation | 255 | 0.93 (0.40–2.13) | 0.860 | 1.44 (0.69–3.00) | 0.326 |
| Rhinitis (18y) | Sex | 255 | 2.19 (1.56–3.07) | <0.001 | 2.54 (1.84–3.49) | <0.001 |
| Rhinitis (18y) | Sex + HDM | 255 | 2.02 (1.44–2.83) | <0.001 | 1.97 (1.34–2.89) | <0.001 |
| Rhinitis (18y) | Sex + polysensitisation | 255 | 1.27 (0.86–1.86) | 0.224 | 1.19 (0.83–1.70) | 0.338 |
| Asthma (26y) | Sex | 255 | 2.98 (1.44–6.17) | 0.003 | 4.11 (2.05–8.24) | <0.001 |
| Asthma (26y) | Sex + HDM | 255 | 2.45 (1.17–5.15) | 0.018 | 3.09 (1.42–6.69) | 0.004 |
| Asthma (26y) | Sex + polysensitisation | 255 | 1.57 (0.67–3.70) | 0.301 | 1.78 (0.72–4.35) | 0.209 |
| Rhinitis (26y) | Sex | 255 | 1.63 (1.27–2.10) | <0.001 | 1.33 (0.92–1.92) | 0.127 |
| Rhinitis (26y) | Sex + HDM | 255 | 1.49 (1.15–1.93) | 0.002 | 1.15 (0.79–1.69) | 0.455 |
| Rhinitis (26y) | Sex + polysensitisation | 255 | 1.13 (0.87–1.47) | 0.370 | 0.82 (0.59–1.14) | 0.234 |

Supplementary Table 11. Risk ratios (RRs) with 95% confidence intervals (CIs) were estimated using Poisson regression with robust standard errors. Models were adjusted for sex alone (primary), and additionally adjusted for house dust mite (HDM) sensitisation at the corresponding age (18 or 26 years) or polysensitisation burden (0, 1, or ≥2 non-*Alternaria* aeroallergen sensitisations) at the corresponding age. These sensitivity analyses use a restricted complete-case subset with complete Alternaria trajectory and confounder data (maximum N=255); effect estimates may therefore differ slightly from the main models due to sample restriction, while direction and inference were consistent. The reference group was the Never trajectory. N denotes the complete-case analytic sample for each model.

Supplementary table 12. Fractional exhaled nitric oxide (FeNO) at 18 and 26 years by *Alternaria* sensitisation trajectory

| *Alternaria* trajectory (10–18–26) | N (18y) | FeNO 18y, GM ppb (95% CI) | N (26y) | FeNO 26y, GM ppb (95% CI) |
| --- | --- | --- | --- | --- |
| Never | 348 | 17.7 (16.3–19.1) | 356 | 15.7 (14.6–16.8) |
| Any | 46 | 24.0 (19.3–29.7) | 43 | 21.4 (17.5–26.2) |
| Persistent | 20 | 26.2 (18.9–36.3) | 21 | 26.2 (19.7–34.9) |

Supplementary table 12. Sex-adjusted geometric mean FeNO (ppb) at 18 and 26 years according to *Alternaria* sensitisation trajectories. Estimates derive from UNIANOVA models of lnFeNO on ordered trajectories and sex, back-transformed to ppb. P for linear trend across trajectories: 18 years p=0.022; 26 years p<0.001.

Supplementary table 13. Sex-adjusted spirometry by *Alternaria* sensitisation trajectory

| Outcome / age | N | Never: mean (95% CI) | Any: mean (95% CI) | Persistent: mean (95% CI) | p for trend |
| --- | --- | --- | --- | --- | --- |
| FEV₁% predicted, 10y | 414 | 104.3 (103.0–105.5) | 104.7 (101.4–108.1) | 102.7 (97.5–107.9) | 0.56 |
| FVC% predicted, 10y | 414 | 103.1 (102.0–104.2) | 104.3 (101.3–107.2) | 102.0 (97.4–106.6) | 0.65 |
| FEV₁/FVC, 10y | 321 | 0.879 (0.872–0.887) | 0.873 (0.855–0.890) | 0.873 (0.846–0.899) | 0.62 |
| FEV₁% predicted, 18y | 425 | 104.7 (103.4–105.9) | 104.4 (101.0–107.8) | 103.0 (97.8–108.1) | 0.53 |
| **FVC% predicted, 18y** | 425 | 102.9 (101.7–104.1) | 103.1 (99.8–106.3) | 102.5 (97.6–107.4) | 0.89 |
| FEV₁/FVC, 18y | 425 | 0.874 (0.866–0.882) | 0.870 (0.850–0.890) | 0.864 (0.834–0.895) | 0.55 |
| FEV₁% predicted, 26y | 432 | 98.1 (96.3–99.9) | 99.3 (94.5–104.2) | 93.6 (86.3–101.0) | 0.25 |
| FVC% predicted, 26y | 432 | 103.2 (101.2–105.1) | 104.5 (99.2–109.8) | 102.0 (93.9–110.0) | 0.78 |
| FEV₁/FVC, 26y | 432 | 0.783 (0.769–0.797) | 0.805 (0.767–0.842) | 0.741 (0.684–0.798) | 0.16 |

Supplementary table 13. Sex-adjusted spirometric indices at 10, 18 and 26 years according to *Alternaria* sensitisation trajectories. Estimates are UNIANOVA marginal means (identity link) with sex and ordered trajectories as predictors. P for linear trend is derived from the polynomial contrast. All analyses use complete-case data per outcome.

Supplementary Table 14. Sex-adjusted log(DRS²) at 10 and 18 years by *Alternaria* sensitisation trajectory

| Age (years) | N | Never: mean log(DRS²) (95% CI) | Any: mean log(DRS²) (95% CI) | Persistent: mean log(DRS²) (95% CI) | p for trend* |
| --- | --- | --- | --- | --- | --- |
| 10 | 320 | 2.12 (1.93–2.31) | 2.34 (1.86–2.81) | 3.22 (2.52–3.92) | 0.003 |
| 18 | 341 | 0.60 (0.43–0.76) | 0.81 (0.34–1.27) | 1.34 (0.68–2.00) | 0.032 |

Supplementary Table 14. Sex-adjusted mean log-transformed dose–response slope (log[DRS²], a marker of bronchial hyper-responsiveness) at 10 and 18 years according to *Alternaria* sensitisation trajectories. Estimates are from UNIANOVA models with ordered *Alternaria* trajectory and sex as fixed effects. p for trend from polynomial contrast across trajectories (Never → Any → Persistent).

1.	Paganelli R, Ansotegui IJ, Sastre J, et al. Specific IgE antibodies in the diagnosis of atopic disease. Clinical evaluation of a new in vitro test system, UniCAP, in six European allergy clinics. *Allergy* 1998; **53**(8): 763–8.
